# MicroRNA Screening Reveals Upregulation of FoxO-Signaling in Relapsed Acute Myeloid Leukemia Patients

**DOI:** 10.3390/genes15121625

**Published:** 2024-12-19

**Authors:** Paula Reichelt, Stephan Bernhart, Uwe Platzbecker, Michael Cross

**Affiliations:** 1 Department of Hematology, Cell Therapy, Hemostaseology and Infectiology, University Hospital Leipzig, 04103 Leipzig, Germany; uwe.platzbecker@medizin.uni-leipzig.de (U.P.); michael.cross@medizin.uni-leipzig.de (M.C.); 2 Interdisciplinary Center for Bioinformatics, Leipzig University, 04107 Leipzig, Germany; berni@bioinf.uni-leipzig.de

**Keywords:** microRNAs, chemoresistance, acute myeloid leukemia

## Abstract

**Background/Objectives**: AML is an aggressive malignant disease characterized by aberrant proliferation and accumulation of immature blast cells in the patient’s bone marrow. Chemotherapeutic treatment can effectively induce remission and re-establish functional hematopoiesis. However, many patients experience chemoresistance-associated relapse and disease progression with a poor prognosis. The identification of molecular determinants of chemoresistance that could serve as potential targets for the therapeutic restoration of chemosensitivity has proven to be challenging. **Methods**: To address this, we have analyzed longitudinal changes in the expression of microRNAs during disease progression in a small set of four AML patients, combined with gene ontology (GO) pathway analysis and evaluation of gene expression data in patient databases. **Results**: MicroRNA profiling of bone marrow samples at diagnosis and after relapse revealed significant differential expression of a large number of microRNAs between the two time points. Subsequent GO pathway analysis identified 11 signal transduction pathways likely to be affected by the differential miRNA signatures. Exemplary validation of the FoxO signaling pathway by gene expression analysis confirmed significant upregulation of *FOXO1* and the target genes *GADD45* and *SOD2*. **Conclusions**: Here, we show how a microRNA-based pathway prediction strategy can be used to identify differentially regulated signaling pathways that represent potential targets for therapeutic intervention.

## 1. Introduction

AML is an aggressive hematopoietic neoplasia characterized by aberrant proliferation and accumulation of immature blast cells in the bone marrow. While the development of novel therapeutic approaches in recent years has improved remission rates, relapse remains a major problem, affecting around half of all patients who achieve remission [1]. It is largely because of this chemoresistance-associated relapse that the 5-year overall survival rate of AML patients is still below 30%. It is, therefore, of the utmost importance to characterize the mechanisms by which AML cells acquire resistance to therapy and to identify druggable targets through which relapse can be either avoided or reverted. One obvious approach to this problem is to search for genes that are expressed differentially between the chemosensitive and chemoresistant stages in a horizontal analysis of successive samples taken from the same patient. However, while mRNA-NGS analyses offer a comprehensive profile of mRNA levels, they take no account of post-transcriptional regulation, which makes a major contribution to determining the level of individual gene products and the biology of both normal and neoplastic hematopoiesis [2]. Since proteomics presents a number of challenges in terms of both accurate quantitation and required sample size, an alternative approach is to focus on post-transcriptional regulation by profiling microRNAs.

MicroRNAs are small, highly conserved, noncoding transcripts of an average of 22 nucleotides in length that regulate gene expression on a post-transcriptional level. They bind specifically to complementary sequences in the 3′ untranslated region (UTR) of their target mRNAs and reduce their translation [3]. By regulating transcription factor levels and modulating feedback or feed-forward loops, microRNAs can have decisive effects on a cell’s entire transcriptional profile [4]. Accordingly, the influences of microRNAs on cellular processes should not be understood as single microRNA-target relations but rather as large regulatory networks controlled by a variety of microRNAs, depending on subcellular location and abundance. MicroRNAs influence physiological hematopoiesis and differentiation processes by a variety of mechanisms [5,6] and can also play a key role in hematopoietic malignancies, where they can serve either as proto-oncogenes or as tumor suppressors depending on the cellular context [7,8,9,10,11]. Indeed, the expression levels of several microRNAs have been found to correlate with the prognosis of AML patients, identifying them as potential biomarkers for acute myeloid leukemia [12,13,14].

The relevance of microRNAs to the specific case of chemoresistance in AML has also been the subject of numerous studies [15,16,17,18]. Importantly, as the complementarity of microRNAs to the target mRNA is limited to a few nucleotides, one microRNA can regulate a large number of targets, while a single mRNA can be regulated by multiple different microRNAs. Furthermore, many microRNAs have been shown to be transcribed in clusters. These characteristics suggest that microRNAs act to orchestrate networks of post-transcriptional regulation. A comparison of microRNA expression profiles between disease states should, therefore, have the potential to highlight coordinated changes in the pathways involved. Indeed, the ways in which microRNA networks influence pathway activity have been a focus of attention over the last decade [19,20,21,22]. Studies of this kind have been facilitated enormously by the rapidly increasing power of computational methods and the accumulation of relevant data in inaccessible databases. The application of modern bioinformatics strategies facilitates the analysis of complex signaling networks and helps reveal how microRNAs mediate crosstalk between signaling pathways. The use of these analytic tools for microRNA-pathway affiliations promises to make microRNA profiles powerful prognostic indicators in the therapy of AML and to aid in the development of new personalized treatment approaches.

Here, we apply microRNA profiling and subsequent pathway analysis to samples from a small set of four selected AML patients who were affected by relapse and chemoresistance. The longitudinal analysis of microRNA signatures in different disease stages combined with analysis of microRNA pathway associations predicted a number of pathways to undergo significant changes during relapse and the acquisition of chemoresistance.

## 2. Materials and Methods

### 2.1. Patients

Samples taken after informed consent from 4 AML patients at diagnosis and relapse were obtained from the University of Leipzig Medical Centre and analyzed in a longitudinal evaluation. Patients included in the analysis did not show primary induction failure but relapsed within a comparatively short time period after remission. None had undergone allogeneic stem cell transplantation between the two sample collection time points. All relevant clinical parameters of the patients are summarized in Table 1.

### 2.2. MicroRNA Expression Profiling

For microRNA profiling, RNA was extracted from bone marrow patient samples using the NucleoSpin miRNA extraction kit (Macherey-Nagel, Düren, Germany) according to the manufacturer’s instructions. To enable statistical evaluation of microRNA expression, RNA extracted from each patient sample was subdivided into 2 aliquots that were subjected separately to NGS, generating 2 technical replicates per sample. For quality monitoring, the integrity of the isolated RNA was confirmed using a fragment analyzer before progressing NGS. RNA integrity control and sequencing were performed by the sequencing core facility of the IZKF Leipzig (Faculty of Medicine, University Leipzig). In total, 10–50 ng of total RNA (RQN 7–10) was used in the small RNA protocol with the NEXTflex Small RNA-seq Kit v3 (Bioo Scientific, Austin, TX, USA). A pool of 12 libraries was used for cluster generation at a concentration of 10 nM using an Illumina cBot (Illumina, San Diego, CA, USA). Sequencing of 50 bp was performed with an Illumina HiScan-SQ sequencer using version 3 chemistry and flowcell according to the manufacturer’s instructions.

### 2.3. Bioinformatics

**Figure 1:**
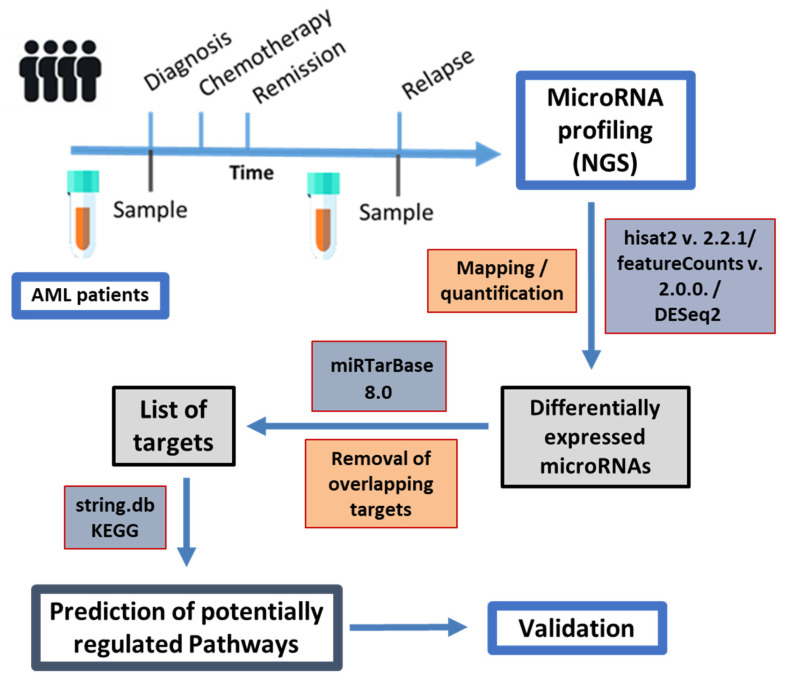
Sampling and workflow for bioinformatic NGS data evaluation and pathway analysis. Paired samples from individual patients taken at diagnosis and after relapse were compared regarding their microRNA expression profiles, which then served as a basis for predicting affected pathways.

Mapping was performed using hisat2 version 2.2.1, mapping read lengths from 15 to 27, with the --passthrough and --wrapper basic-0 options applied against a hg38 assembly with masked tRNAs and microRNAs, where microRNAs and tRNAs were added individually as artificial chromosomes. MicroRNAs were counted using featureCounts version 2.0.0 with the -M option, counting only mature microRNAs using a custom-made gene transfer format (gtf) file containing the location of the mature microRNAs on the respective artificial chromosomes. The single patient data were analyzed using DESeq2. MicroRNAs with an adjusted *p*-value below 0.05 and an absolute log2 fold change greater than 1 were analyzed further. Non-weak targets of these microRNAs from miRTarBase 8.0, as downloaded in July 2021, were filtered for genes that were targeted both by down- and upregulated microRNAs. The resulting genes were analyzed using string.db [23] for KEGG (Kyoto Encyclopedia of Genes and Genomes) and GO (Gene ontology) term enrichment. The significance level for FDR was defined below 0.05. The workflow for sampling, bioinformatics, and validation is depicted in Figure 1.

### 2.4. Reverse Transcription and qRT-PCR

From each RNA sample, 1000 ng were diluted in 11.5 μL RNAse-free water and reverse transcribed into cDNA by means of RevertAid First Strand cDNA Synthesis Kit (Thermo Fisher SCIENTIFIC, Waltham, MA, USA), according to the manufacturer’s instructions, using random hexamer primers. Reverse transcription was performed at 42 °C for 60 min, followed by a termination step at 70 °C for 5 min. cDNA was frozen at −20 °C or directly used for quantification of selected transcripts by means of QuantiTect SYBR Green PCR Kit (Qiagen, Venlo, The Netherlands). U6 snRNA, a key component of the spliceosome, was used as a “housekeeping gene” for all SYBR Green-based RT-qPCR analyses. Relative expression levels and log2 fold changes in target genes in 2 samples were calculated according to the delta-delta-CT method.

### 2.5. Statistical Analyses

Statistical significance between expression levels measured in samples taken at diagnosis and after relapse was determined by Student’s *t*-test, where data were normally distributed. *p*-values < 0.05 and <0.01 were considered significant and highly significant, respectively. For TCGA database analysis, patients were subdivided by risk groups, or p53 mutation status and mRNA expression data were tested for normalcy and equal variance using Shapiro–Wilk and Levene tests. Finally, significant differences within the groups were analyzed using the Kruskal–Wallis and Dunn test. For overall survival plots, patients were subdivided into high and low expression by their median. Kaplan–Meier plots and statistics were generated using the survminer package in R studio software [24].

## 3. Results

### 3.1. Relapse and Treatment Failure Are Associated with Fundamental Changes in the microRNA Signature of AML Patients

**Figure 2:**
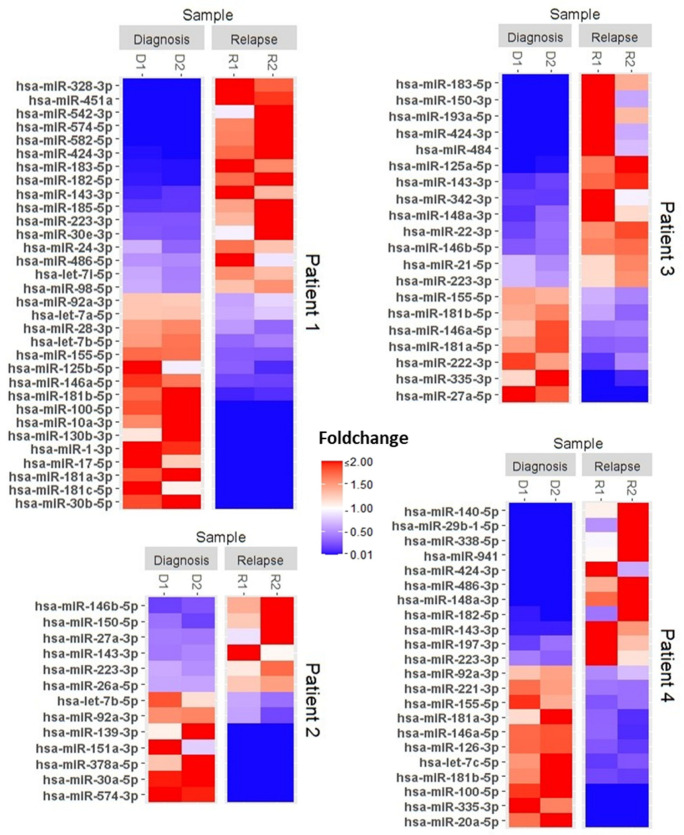
Relapse is associated with significant changes in individual microRNA signatures. MicroRNA expression profiles generated by next generation sequencing for microRNAs of individual AML Patients at diagnosis and relapse. Only significantly differential expressed microRNAs are shown. Data from 2 technical replicates for each patient are presented as expression levels relative to the mean of all four levels measured for each microRNA. Red represents increased and blue decreased expression.

For each patient, individual microRNA expression profiles were determined based on the bioinformatic evaluation of read counts in the two technical replicates for each sample. MicroRNA profiling indicated significant differential expression of varying numbers of microRNAs prior to versus post-relapse in each of the four patients (Figure 2).

As expected, the composition and number of differentially expressed microRNAs differed widely between the four cases assessed here. Indeed, given a sufficiently large cohort of matched samples, a personalized approach to individual microRNA profiling for single patients is likely to reveal complex associations between microRNA expression and a range of variable clinical patient characteristics. More relevant for our small cohort was the observation that a subset of microRNAs was consistently differentially expressed in multiple patients, suggestive of commonalities in up- or downregulation in relapse. This was the case for the microRNAs miR-223-3p, miR-92a-3p, miR-143-3p, miR-146a-5p, and miR155-5p.

**Figure 3:**
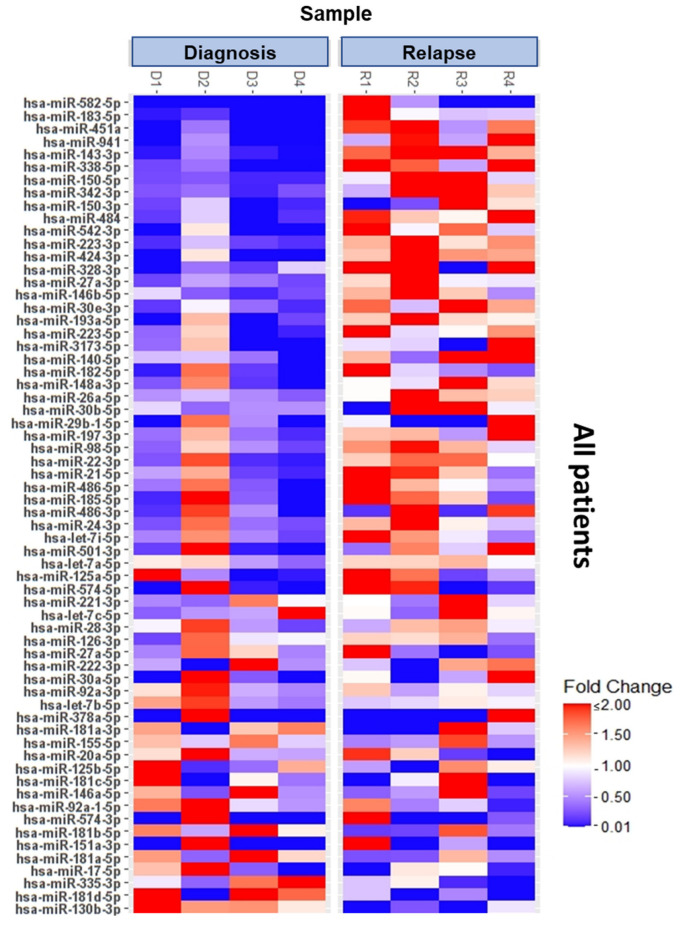
MicroRNA profiling reveals expression changes in a large variety of microRNAs in patients after AML relapse. Overall analysis of significantly differential expressed microRNAs in AML patients at diagnosis and relapse. Data summarize four patients representing biological replicates while each colored box marks the relative expression of the indicated miRNA in comparison to the corresponding control. Red represents increased and blue decreased expression.

To extend the individual patient analysis, statistical analysis was performed using data from all four patients to determine significantly differentially expressed microRNAs in relapse compared to diagnosis. Figure 3 shows a heat map that includes all microRNAs undergoing significant changes in expression level in the overall analysis. The large number of microRNAs undergoing significant shifts in expression confirms that relapse and treatment failure are indeed associated with fundamental changes in the microRNA signature of AML patients.

### 3.2. MicroRNA Profiling Indicates FoxO-Signaling to Be Associated with Relapse and Chemoresistance

**Figure 4:**
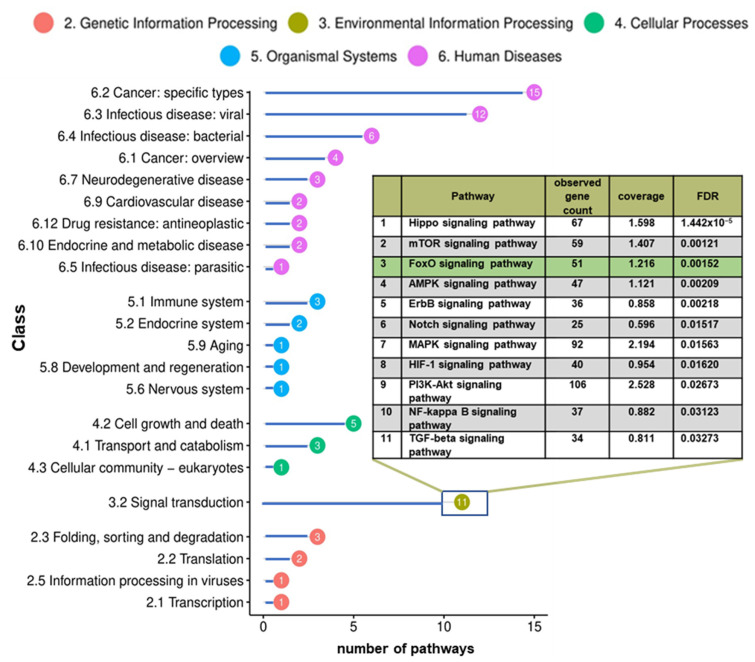
Pathway analysis predicts FoxO signaling to be affected by differential microRNA expression in chemoresistant AML. MicroRNA profiling based gene ontology pathway analysis in total predicted 83 KEGG signaling pathways to undergo significant activity alterations between the time of diagnosis and that of relapse. Within the KEGG category of “signal transduction”, 11 pathways were indicated.

Coordinated changes in the level of multiple microRNAs that target the same pathway imply changes in that pathway to be potentially associated with chemoresistance. To determine if the observed changes in microRNA expression levels correlate to specific pathway regulations, GO term analyses were carried out based on microRNA target associations. This examination predicted 82 KEGG pathways to be affected significantly by changes in microRNA expression. Most of the pathways so identified are classified in the KEGG area of “human diseases” (Figure 4). An overview of all predicted pathways is provided in Supplementary Table S1.

The identification of pathways with altered activity in relapse can serve to identify potential molecular targets for new therapy approaches to chemoresistant AML. As the large number of over 80 pathways was too comprehensive for detailed evaluation, further analysis was focused on the KEGG class “signal transduction”, as these tend to be the most amenable targets for activity modulation by chemical compounds. Within this category, 11 pathways were predicted to be impacted by microRNAs differentially expressed between diagnosis and relapse. Figure 4 shows these ranked by FDR. Strikingly, the TGF-β pathway that we have previously identified and validated by applying a similar approach to a cell line model of drug resistance was also indicated to be associated with drug resistance and relapse in the microRNA analysis of primary AML [25]. This suggests that at least some key associations between microRNA signature and chemoresistance apply similarly to both cell line models and to primary patient AML cells.

The signaling cascade predicted to have the lowest FDR was the Hippo signaling pathway. This evolutionarily conserved pathway regulates tissue growth and organ size in a wide range of species, including humans. Hippo signaling has been implicated in many types of human cancer [26,27,28,29,30], including leukemia [31,32,33], although the relevance to chemoresistance remains unclear. Furthermore, its role in solid tumors has been more evident.

The mTOR (mammalian Target of rapamycin) pathway, with the second lowest FDR, is a central regulator of survival, proliferation, and immune cell differentiation. MTOR signaling plays an important role in apoptosis, autophagy, and tumor metabolism and, as such, is highly interconnected to other pathways identified here, such as PI3K-Akt, MAPK, or AMPK signaling [34]. As changes in PI3K-Akt-mTor signaling are already known to be associated with AML and have recently been linked to chemoresistance, inhibition of PI3K-Akt-mTOR is already considered to be a potential therapeutic strategy in AML [35,36,37,38].

The FoxO signaling pathway, which was identified here with a predicted FDR very similar to that of mTOR, has also been associated with chemoresistance in a variety of tumor entities. However, the role of FoxO in AML relapse and chemosensitivity has not been elucidated in great detail. As FoxO-signaling was also indicated by microRNA profiling to play a role in chemosensitivity in a cell culture model for chemoresistance it was chosen here for further experimental evaluation. This pathway regulates the activity of growth factor and stress-dependent FoxO transcription factors involved in the clearance of reactive oxygen species, DNA damage repair, and cell cycle control. The transcription factors FoxO1 and FoxO3, in particular, have been linked to the emergence and progression of a variety of cancer entities and suggested as therapeutic targets [39,40].

Evaluation of the data from primary AML can predict the involvement of differentially regulated pathways but does not directly predict whether pathway activity increases or decreases in chemoresistance. In an attempt to provide an indication of the direction of change, all predicted FoxO pathway targets of the differentially expressed microRNAs were analyzed. As shown in Supplementary Table S2, only one of the differentially expressed microRNAs (miR-223-3p) is predicted to target a FoxO mRNA (FoxO1) directly. This was upregulated in relapse, which would be consistent with the down-regulation of FoxO1. However, since this concerns only a single predicted interaction, it is not a robust indication. All other microRNAs that were up or downregulated in relapse and impact the FoxO pathway are predicted to target mRNAs coding for signaling proteins that can modulate the activity of FoxOs, such as PTEN, Nemo-like kinase (NLK), or MAP kinases. As these signaling proteins can act either positively or negatively on FoxO signaling, the list of targets alone does not indicate the overall direction of the regulation.

### 3.3. Gene Expression Analysis Reveals Significant Shifts in FoxO Signaling Activity in Patients with Relapsing AML

**Figure 5:**
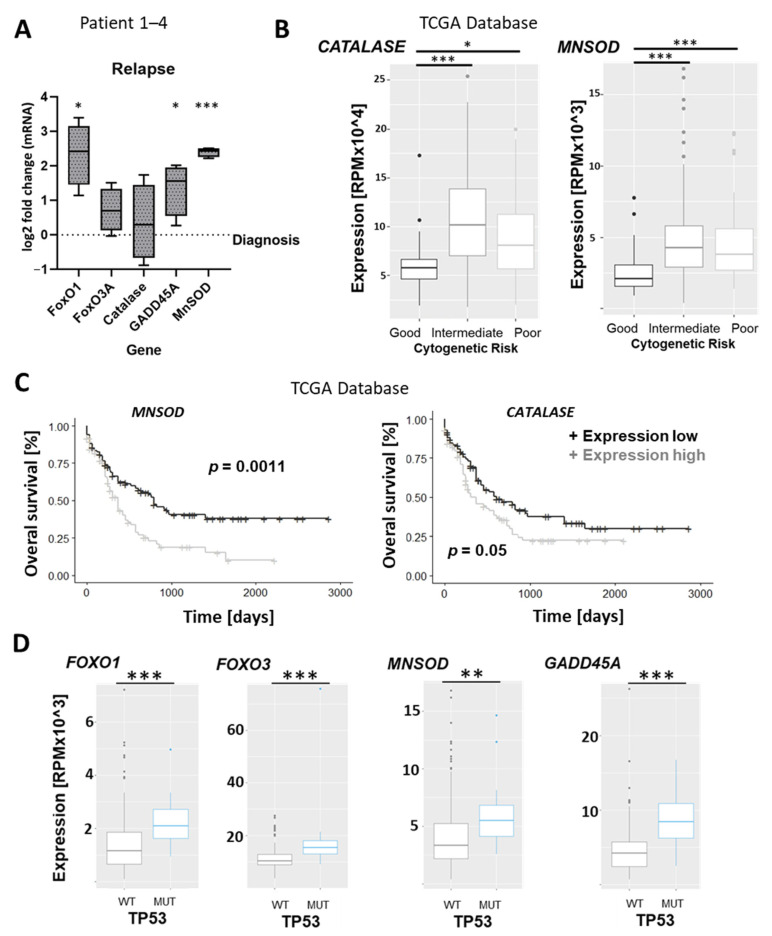
FoxO signaling is upregulated in relapsed AML and is associated with mutations in the tumor suppressor p53 gene; (**A**) expression of FoxO signaling pathway genes in relapsed AML patients (bars represent log2 fold change normalized to values at diagnosis). The horizontal lines show the medians, box limits indicate the 25th and 75th percentiles as determined by R software, and whiskers extend to minimum and maximum values. (**B**) Expression of FoxO signaling target genes Catalase and *MNSOD* in different cytogenetic risk groups, according to the MRC (the British Medical Research Council). Center lines show the medians, box limits indicate the 25th and 75th percentiles as determined by R software, whiskers extend to the 5th and 95th percentiles, and outliers are represented by dots. (**C**) Overall survival of AML patients (TCGA Research Network), according to the median divided by *MNSOD* and Catalase expression. *p*-values were calculated using the Log-rank test (R software). (**D**) Gene expression of FoxO transcription factors and target genes in AML patients with and without P53 mutations. Statistical significance is indicated as * *p* ≤ 0.05, ** *p* ≤ 0.01, *** *p* ≤ 0.001.

The validation of changes in FoxO pathway activity is made challenging firstly by the limited availability of suitable patient material for reliable protein analysis and secondly by the fact that microRNAs can affect protein translation levels without changing the level of the corresponding target mRNA with which they interact, obviating validation of predicted miRNA targets by RT-PCR. For these reasons, changes in FoxO pathway activity were studied by assessing the expression levels of the genes that it ultimately affects. As central regulators of the cellular oxidative stress response, FoxO proteins directly control the transcription of genes involved in the clearance of reactive oxygen species, including superoxide dismutase (*MNSOD*) and catalase. The DNA repair gene *GADD45A* is also directly induced by FoxO members and was also chosen for targeted analysis, as were the *FOXO1* and *FOXO3* genes themselves. Assessment of gene expression at the mRNA level revealed significant upregulation of *FOXO1*, *GADD45A*, and *MNSOD* in the relapse samples compared to the diagnosis time point (Figure 5A). The most significant difference could be observed for *MNSOD*, which was robustly upregulated in all four patient samples. To assess whether the observation that FoxO signaling is significantly upregulated in patients facing relapse and chemoresistance is applicable to a larger cohort of patients, we used the AML patient dataset of the cancer genome atlas (TCGA) for gene expression analysis. Although the wide coverage of the TCGA provides comprehensive gene expression and clinical patient data, the database does not allow longitudinal comparisons of individual patients over different disease stages. We, therefore, looked for associations between FoxO target gene expression and both cytogenetic risk group and overall survival, in which chemoresistance and relapse play a major role. This revealed a significantly lower expression of the FoxO target genes Calatase and *MNSOD* in AML patients with a favorable prognosis (Figure 5B), while high expression of *MNSOD* was associated with a worse overall survival (Figure 5C). The same trend could be observed for Catalase but did not reach significance.

Finally, we looked for a relationship between FoxO target gene expression and mutations in the tumor suppressor gene P53 since these define a subset of AMLs with a particularly poor prognosis and high rates of chemoresistance and relapse. Interestingly, we found significantly higher expression levels of *FOXO1*, *FOXO3*, and FoxO target genes *MNSOD* and *GADD45A* in P53 mutated patients (Figure 5D). Taken together, this database analysis supports the wider clinical relevance of our experimental findings by indicating a high activity of FoxO transcription factors in patients at high risk for chemoresistance and relapse.

## 4. Discussion

The development of chemoresistance and associated relapse significantly reduces the therapeutic options for AML patients. Accordingly, the restoration or prolongation of chemosensitivity has the potential to improve survival and quality of life and to provide a bridge to stem cell transplantation in eligible patients. Here, we report the use of microRNA expression profiling to identify pathways mis-regulated in chemoresistance. This could be a first step to finding potential targets for new strategies to avoid or reverse chemoresistance.

The profiling of microRNAs investigates gene regulation at the post-transcriptional level, which is now known to make a decisive contribution to differential gene expression [41,42,43]. In this sense, microRNA profiling can be considered to be a complementary alternative to transcriptomics. A feature of the microRNA approach is the focus on a relatively small, interactive network composed exclusively of regulators with predictable targets. Each regulator may target multiple targets, and each target may be affected by multiple regulators. It should, therefore, be feasible to sample cell phenotype via a relatively small number of variables.

Using this approach, fundamental changes in microRNA signatures could clearly be observed in the bone marrow of four individual patients between pretreatment and relapse stages. The sample size was limited by the rarity of longitudinal sample sets from relevant disease stages of the same patient since many patients who are suitable for ARA-C-based chemotherapy proceed to hematopoietic stem cell transplantation after reaching remission. Despite this limitation, an overarching analysis revealed consistent patterns of differential expression for single microRNAs. Analysis of the predicted targets of differentially expressed microRNAs implied the involvement of specific processes and pathways in chemoresistance, including the TGF-β pathway that we have recently found to be implicated in chemoresistance in a cell line model [25]. Among a number of other pathways implicated in this way, we chose the FoxO pathway for further investigation since it acts as a hub that integrates multiple inputs to control processes such as apoptosis, DNA repair, and cell cycle that are likely to be of high relevance to chemoresistance. Indeed, the toxicity of many prominent chemotherapeutic drugs has previously been shown to be FoxO-mediated [44,45,46,47].

Although microRNAs typically decrease the expression of their targets, it is not possible to predict the balance of expression in a complex pathway in which both positive and negative regulators may be affected. However, our analysis of both FoxOs and their target genes at the mRNA level suggests strongly that FoxO pathway activity is lowest in AML patients with favorable risk, higher in higher risk patients, and increases following relapse, suggesting that chemoresistance is accompanied by an increase in FoxO signaling. Whether or not FoxO contributes in a decisive way to the emergence of chemoresistance or is induced as a consequence remains unclear and will require more detailed investigation. Specifically, the consistently high expression of FoxO pathway genes in P53mut AML may be a result rather than a cause of the more aggressive disease. However, given the role of FoxO in DNA damage repair, the induction of apoptosis, and block in cell cycle progression, it seems likely that increased activity may be induced in cells stressed by survival and proliferation under chemotherapy. In this case, selective reduction in FoxO signaling in these cells may have the potential to reinstate at least a degree of chemosensitivity.

In summary, the longitudinal analysis of microRNA signatures in different disease stages combined with analysis of microRNA-pathway associations predicted a number of pathways to undergo significant changes during relapse and the acquisition of chemoresistance. Changes in the expression of pathway components at the mRNA level were validated by targeted molecular analysis and also by a broad analysis of gene expression data available in the Cancer Genome Atlas database. While it remains unclear whether the observed changes contribute causally to chemoresistance or occur as a downstream consequence of progression, the approach described here successfully identifies pathway changes associated with changing disease states.

## Figures and Tables

**Table 1:** Patient characteristics.

Patient Nr.	Sex	Age at Diagnosis	Karyotype	Cytogenetic Risk	Days Until Relapse	Mutation Profile
1	w	**59**	46 XX	favorable	208	NPM1mut Typ A, CEBPAwt, FLT3-ITD low ratio, FLT3 TKDwt, IDH1 R132C, IDH2wt, DNMT3A R882wt
2	w	**57**	46 XX	favorable	266	biall CEBPAmut, NPM1wt, FLT3-ITD low ratio, FLT3-TKD, IDH1 and 2 wt
3	m	**38**	46, XY, del(9)(q21q32)[2]&46, XY [29]	favorable	113	biall CEBPAmut, NPM1wt, FLT3-ITD wt, FLT3-TKD wt
4	w	**35**	46, XX	intermediate	175	CEBPAwt, NPM1wt, FLT3-ITD wt, FLT3-TKD wt, IDH1 and 2 wt, DNMT3A wt

## Data Availability

The materials described in this manuscript, including all relevant raw data, will be freely available to any researcher wishing to use them for non-commercial purposes without breaching participant confidentiality. Datasets generated during and/or analyzed during the current study are available from the corresponding author upon reasonable request.

## Supplementary Materials

The following supporting information can be downloaded at https://www.mdpi.com/article/10.3390/genes15121625/s1, Table S1: Overview of all predicted pathways by GO term analysis, Table S2: Overview of all predicted FoxO signaling targets.
